# Chronic Total Occlusion of the Left Main Coronary Artery With Preserved Left Ventricular Systolic Function Presenting as a Chronic Coronary Syndrome: A Case Report and Brief Review

**DOI:** 10.7759/cureus.46830

**Published:** 2023-10-11

**Authors:** Sidar Şiyar Aydın

**Affiliations:** 1 Cardiology, Erzurum City Hospital, Erzurum, TUR

**Keywords:** coronary artery bypass grafting (cabg), chronic total occlusion, left main coronary artery disease (lmcad), coronary collateral circulation, left main coronary artery disease

## Abstract

The incidence of lesions involving the left main coronary artery (LMCA) during coronary angiography is approximately 5% to 8%. It usually presents with acute coronary syndrome and can be fatal. Total occlusion of the LMCA is rare, often accompanied by myocardial infarction and cardiogenic shock. We present an LMCA chronic total occlusion case in a 60-year-old female patient with chronic coronary syndrome. In our case, the LMCA was selectively visualized, and it was found to be occluded. The right coronary artery fed the entire left system through the collateral network. The patient had no risk of coronary artery disease other than hypertension. Successful coronary artery bypass grafting was performed without any complications.

## Introduction

The incidence of lesions involving the left main coronary artery (LMCA) during coronary angiography is approximately 5% to 8% [1]. It is usually seen together with multi-vessel disease, often presents with acute coronary syndrome, and may have a fatal course. However, LMCA chronic total occlusion (CTO) is extremely rare, and its prevalence cannot be estimated exactly [2]. Coronary artery bypass grafting (CABG) is still the recommended treatment strategy [3].

In this case, we present a 60-year-old female patient with chronic coronary syndrome with preserved left ventricular systolic functions, which was detected as LMCA CTO on coronary angiography and then treated with CABG.

## Case presentation

**Figure 1:**
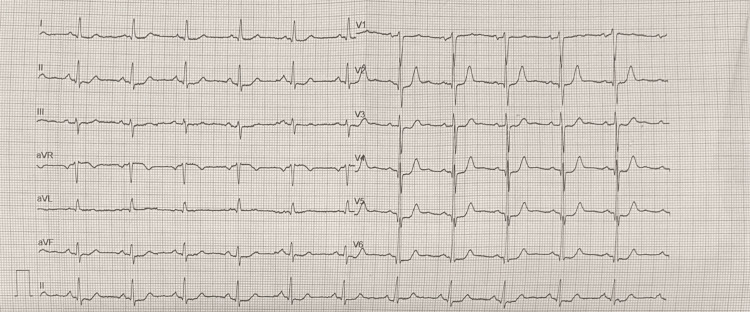
The patient’s 12-lead electrocardiography at the time of initial admission.

A 60-year-old postmenopausal female patient presented with the complaint of angina pectoris for about six months. The patient had a history of hypertension (HT). There was no history of smoking, fever, arthralgia or arthritis, chronic kidney disease, diabetes mellitus, radiation therapy, or myocardial infarction. Electrocardiography revealed left ventricular hypertrophy (LVH) and left axis deviation (Figure 1).

**Figure 2:**
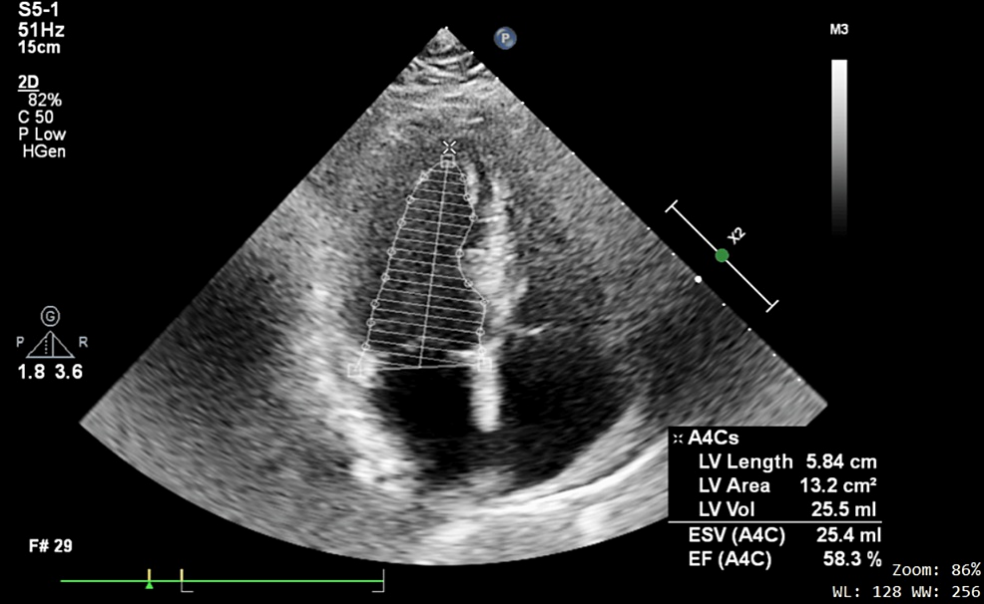
Transthoracic echocardiographic image showing normal left ventricular systolic function.

On two-dimensional transthoracic echocardiography, the ejection fraction was 58.3%, and stage 1 diastolic dysfunction and LVH were detected. No signs of heart valve disease were found (Figure 2).

**Figure 3:**
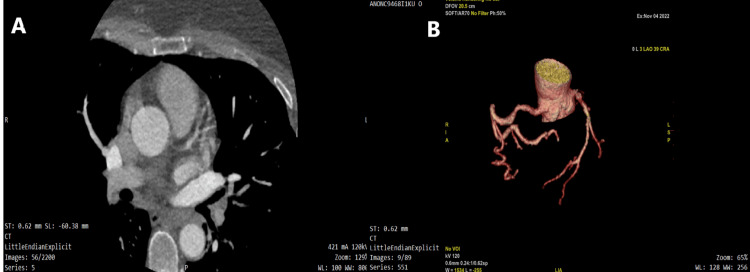
A and B: Multidetector coronary computed tomography images.

Troponin I was negative, low-density lipoprotein cholesterol was 89 mg/dL, high-density lipoprotein was 42 mg/dL, and triglyceride and total cholesterol were within normal limits on blood tests. The patient’s liver and kidney functions were found to be normal. Multidetector coronary computed tomography (MDCT) was planned for the patient. As severe stenosis in the LMCA and the right coronary artery (RCA) were reported in the MDCT, diagnostic coronary angiography and revascularization were planned, if appropriate and indicated (Figures 3A, 3B).

**Figure 4:**
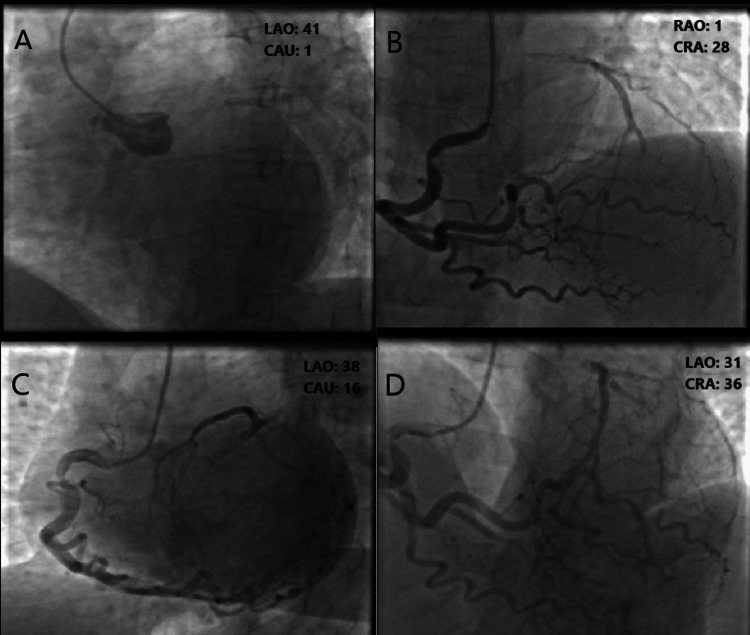
A: Angiographic view of chronic total occlusion of the left main coronary artery. B, C, and D: Severe lesion in the right coronary artery with the left system supplied with blood from the right coronary artery using collaterals. LAO: left anterior oblique; RAO: right anterior oblique; CAU: caudal; CRA: cranial

A transradial approach was applied to the patient with a 6F sheath. Then, 5,000 IU of heparin was administered. A 6F 4.0 left Judkins catheter (Shunmei, The Hague, Netherlands) was applied to the LMCA selectively, and the LMCA was found to be 100%. Subsequently, the RCA was cannulated with a right 6F 4.0 Judkins catheter (Shunmei, The Hague, Netherlands), and critical stenosis was detected in the RCA proximally (Figures 4A, 4B). It was observed that the RCA filled the left system up to the LMCA ostium through Rentrop grade 3 collaterals connected to the left descending coronary artery (LAD) and circumflex branches. In the left cranial and left caudal angulation, it was observed that the left system was completely fed through the RCA (Figures 4C, 4D).

The patient’s hemodynamic parameters were stable during the procedure, and she did not have angina. The patient was evaluated in the cardiology and cardiovascular surgery council. CABG was recommended to the patient. Upon the patient’s acceptance of the procedure, she was transferred to surgery. After a successful CABG procedure, the patient was discharged with acetylsalicylic acid 100 mg, clopidogrel 75 mg, metoprolol, atorvastatin, a combination of angiotensin-converting enzyme inhibitor, and indapamide.

## Discussion

LMCA occlusion is usually rare. While acute occlusion of the LMCA presents with anterior myocardial infarction, pulmonary edema, and cardiogenic shock, cases with chronic occlusion usually present with symptoms of angina [4,5]. Considering the previous studies, LMCA occlusion occurs in the 14-87-year age range. LMCA lesions are usually located in areas containing bifurcation regions. It is seen in women and young people with fewer atherosclerotic risk factors [6]. In-hospital mortality in LMCA occlusions is not correlated with age [7]. Our case was older than the cases described in the literature. While familial dyslipidemia and Kawasaki are blamed in young people, atherosclerotic plaque rupture is blamed more frequently in older patients. Apart from atherosclerosis, spontaneous coronary artery dissection, iatrogenic injury, rheumatoid arthritis, syphilis, radiation therapy, and arthritis may also be etiological reasons [3,7].

The prevalence of LMCA CTO lesions is thought to be 0.04%. However, its true prevalence may be difficult to estimate, as it causes out-of-hospital sudden cardiac death [8]. Survival of LMCA occlusion depends on RCA dominance, size, and development of the collateral network [9]. Almost half of the cases have an RCA lesion. In a study involving seven patients with LMCA CTO, RCA lesions were detected in three patients, and the left ventricular systolic functions of these patients were found to be impaired [10]. However, in our case, although the patient had a critical RCA lesion, left ventricular systolic functions were preserved.

The collateral classification used angiographically evaluates the filling strength of the vascular bed distal to the CTO rather than the collateral network [11]. In cases of LMCA CTO, it may be difficult to visualize the filling, mostly from the RCA to the distal LMCA, based on the diameter and flow rate of the collateral vascular bed. In our case, the LMCA was clearly visualized. In a review of three LMCA CTO cases, angina pectoris was associated with the strength of the collateral connection [12]. In the literature, cases with critical stenosis or CTO in the LMCA can usually experience symptoms even with the slightest effort or at rest. It may be accompanied by resting angina, exertional or resting dyspnea, and signs of heart failure [13]. Because LMCA occlusion usually presents with acute coronary syndrome, often accompanied by cardiogenic shock, mechanical support may be needed, and urgent revascularization is required [14]. Our case did not present with acute coronary syndrome, and her hemodynamics were stable. Unlike other cases, there was no resting angina, preserved systolic functions, and no signs of heart failure. This situation can be associated with the long and chronic spread of the atherosclerotic process of the patient and the strong collateral vascular network that develops accordingly. In addition, a study conducted among patients with acute myocardial infarction found that adverse outcomes, including heart failure, were less common in the presence of angiographic collateral extending to the responsible lesion [15]. A study including CTO patients found that well-developed collateral connectivity was associated with better myocardial viability and left ventricular systolic function [16].

CABG is considered a leading treatment strategy in cases of LMCA CTO [17]. However, due to technical and hardware-related advances in percutaneous coronary intervention, percutaneous coronary intervention may be an alternative treatment in cases where the left system is protected (previous CABG) and there is a strong collateral network [18]. However, percutaneous coronary intervention is preferred more frequently in older age groups and patients with low and medium SYNTAX scores [19]. Considering our patient’s age, SYNTAX score, and relatively young age, CABG was preferred as the treatment strategy.

## Conclusions

LMCA occlusions are rare and usually present in acute cases. Patients presenting with chronic symptoms do not exclude LMCA occlusion, and good collateral may preserve left ventricular systolic functions and hide the symptoms and severity of the disease. While atherosclerosis is the main cause, other causes should be carefully examined. Although CABG remains the first choice for the revascularization strategy, percutaneous coronary intervention may be considered in appropriate cases.
